# Varying Estimates of Sepsis among Adults Presenting to US Emergency Departments: Estimates from a National Dataset from 2002-2018

**DOI:** 10.1177/08850666221080060

**Published:** 2022-02-28

**Authors:** Sriram Ramgopal, Christopher M Horvat, Mark D Adler

**Affiliations:** 1 Northwestern University Feinberg School of Medicine, Chicago, IL, USA; 2 12317 University of Pittsburgh School of Medicine; UPMC Children’s Hospital of Pittsburgh, Pittsburgh, PA, USA; 3 UPMC Children’s Hospital of Pittsburgh, Pittsburgh, PA, USA

**Keywords:** sepsis, emergency medicine, septic shock, severe sepsis

## Abstract

**Background:**

A variety of approaches to defining sepsis using administrative datasets have been previously reported. We aimed to compare estimates, demographics, treatment factors, outcomes and longitudinal trends of patients identified with sepsis in United States emergency departments (EDs) using differing sets of sepsis criteria.

**Methods:**

We performed a cross-sectional study using the National Healthcare Ambulatory Medical Care Survey, a complex survey of nonfederal US ED encounters between 2002 to 2018. We obtained survey-weighted population-adjusted encounters of sepsis using the following criteria: explicit sepsis, severe sepsis, and quick Sequential Organ Failure Assessment (qSOFA) score combined with the presence of infection.

**Results:**

Age-adjusted for US adults, 18.6, 16.1 and 8.9 encounters per 10 000 population were identified when using the explicit, severe sepsis and qSOFA definitions, respectively. A higher proportion of the explicit cohort was hospitalized and had blood cultures performed, compared to cohorts ascertained using severe sepsis and qSOFA criteria, though confidence intervals overlapped. Antibiotic use was highest in encounters meeting qSOFA criteria. When inspecting unweighted encounters meeting each set of criteria, there was minimal overlap, with only 3% meeting all three. Encounters meeting the explicit and severe sepsis criteria were increasing over time.

**Conclusion:**

The explicit, severe sepsis and qSOFA criteria generated similar annual rates of presentation when applied to US ED encounters, with some evidence of the explicit sepsis cohort being higher acuity. There was minimal overlap of cases and instability in estimates when assessed longitudinally. Our findings inform research efforts to accurately identify sepsis among ED encounters using administrative data.

## Introduction

The definition of sepsis has undergone substantial modification in the last three decades. Earlier systemic inflammatory response syndrome (SIRS) criteria were based on vital sign and laboratory abnormalities, combined with suspicion for infection.^1^ Clinical definitions for sepsis have undergone substantial evolution in the ensuing three decades with the recent introduction of the Sepsis-3 criteria.^2,3^ Concurrently, there have been updates to research criteria to identify sepsis using administrative or other large datasets.^4–7^ A variety of approaches have been proposed to identify sepsis in such data sources, including methods identifying organ dysfunction coinciding with infection for severe sepsis, as well as with the use of explicit diagnosis codes. Research criteria for sepsis, which often rely on administrative and claims data for cohort ascertainment, are crucial to obtain population-level estimates of sepsis and to help understand the overall morbidity and mortality of this disease. Such criteria carry implications with respect to public health, policy, and quality improvement.^8^

Several recent studies have reported on the epidemiology of adults sepsis seen in the emergency department (ED) when using a variety of acquisition methods.^5,6,9–11^ However, many of these publications have been limited to the ICD-9 era in the United States and did not directly compare differences in acuity and overlap between sepsis definitions, a finding which is critical to establish a consensus for these criteria for epidemiological purposes. In this investigation, we therefore aimed to compare estimates, demographics, treatment factors and outcomes of patients identified with sepsis when using multiple criteria applied to a national administrative dataset for ED encounters from the United States.

## Methods

### Data Source

We performed a repeated cross-sectional analysis of the National Hospital Ambulatory Medical Care Survey (NHAMCS), a nationally representative cross-sectional probability sample survey of visits to EDs.^12^ NHAMCS is conducted annually by the Centers for Disease Control and Prevention National Center for Health Statistics (NCHS). Each record (ie “count”) is de-identified and assigned a weight equal to the inverse of its probability of being included in the sample.^13^ The survey operates at four stages: (1) county-level primary sampling units, (2) hospitals within primary sampling units, (3) EDs within hospitals, and (4) patient visits within EDs.^14^ The weights within the dataset can be used by statistical packages to extrapolate survey-weighted population estimates with standard errors for US ED encounters. Research performed using NHAMCS is approved by NCHS Ethics Review Board. Common data elements may be combined for the purposes of increasing sample size. We used data for the period 2002–2018 for the present study. Our study population was limited to adults (≥18 years).

### Sepsis Criteria

A number of criteria have been used to identify patients with sepsis from large datasets. These criteria include “explicit” criteria which rely on direct coding of a diagnosis for sepsis or septic shock;^6,11,15–17^ “severe sepsis” criteria, which rely on the combined presence of infection and organ dysfunction to identify patients,^4,5,15,17^ assessment of vital sign irregularities to identify patients meeting criteria for systemic inflammatory response syndrome in the presence of suspected infection,^7,18,19^ elevated Sequential Organ Failure Assessment (SOFA) or quick Sequential Organ Failure Assessment (qSOFA) score in the presence of suspected infection,^15,18,19^ or treatment-based criteria in which specific treatment factors are used to identify candidate patients. For the present investigation, we evaluated three sepsis criteria that have been previously described in the literature for administrative datasets: an explicit sepsis criteria, a severe sepsis definition introduced by Angus and refined by Wang, and a qSOFA score combined with the presence of infection.^4–6,9,20^ The explicit, severe sepsis and qSOFA criteria have been widely used in administrative datasets (including NHAMCS) to identify patients with sepsis.^5,6,9^ Our definition of severe sepsis was based on the combined presence of organ dysfunction with suspicion for infection, a pairing sometimes referred to as “implicit” sepsis. We included the qSOFA score as an alternative to explicit and severe sepsis definitions as it is a commonly used bedside method to screen adults with sepsis in the ED, and because of prior work supporting a role for the qSOFA score in predicting adverse outcomes in patients with sepsis in the intensive care unit^21^ and the ED.^22,23^

A summary of the definitions used as applied to the NHAMCS dataset is provided in Supplementary Table 1. We assessed diagnosis codes using the International Classification of Disease, ninth revision and tenth revision (ICD-9 and ICD-10) codes. We converted ICD-9 codes to ICD-10 using Generalized Equivalence mapping, a resource provided by the Centers for Medicare and Medicaid resources that provides bidirectional cross-walking of ICD codes.^24^ Diagnoses were determined using ICD-9 and 10 codes assigned in the ED and during subsequent hospitalization (from 2005 onwards) when available. Because of limitations with the availability of the Glasgow Coma Scale (GCS) within NHAMCS, the qSOFA criteria were applied only for the years 2009 to 2011. Any inclusion of vital sign abnormalities utilized those assessed at triage.

### Data Acquisition

We extracted encounter demographics, assessment, diagnostic testing, treatment and clinical outcome data. Demographics included age (by intervals of 18-64 and ≥65 years), sex, race (classified in the NHAMCS dataset as White, Black, and other), ethnicity, insurance status (private, public, and other), hospital arrival mode by emergency medical services (EMS), and location in a metropolitan status area. Clinical assessment data included temperature, heart rate, respiratory rate, and systolic blood pressure (classified as abnormal, normal, or missing SIRS criteria).^1^ Diagnostic testing included blood culture, complete blood count, lactate, urine culture, urinalysis, and radiography. Treatment factors included the provision of antibiotics, vasoactive agents, intravenous fluids, the performance of endotracheal intubation, disposition and in-hospital mortality. NHAMCS contains dedicated fields for procedures of intravenous fluid provision and endotracheal intubation. Disposition was classified from the NHAMCS dataset into groups of admitted/transferred, discharged, in-hospital mortality, and all others.

### Analysis

We calculated crude and age-adjusted rates of each type of sepsis presentation. We assessed overlap among unweighted encounters by constructing a Euler diagram for the years when all three criteria could be computed (2009-2011). We reported estimates using survey-weighting procedures accounting for the NHAMCS sampling design. We reported yearly trends in sepsis diagnosis adjusted for the adult population using the American Community Survey.^25^ In keeping with guidelines established by NCHS, estimates with fewer than 30 records or with a relative standard error greater than 30% were considered unstable and not reported.^26^ Demographics were provided as estimates or percentages, using 95% confidence intervals (CI).

We performed two *post-hoc* analyses. First, we compared cohorts of patients with explicit sepsis and severe sepsis from the years from 2016 to 2018 as this would be more representative of recent care practices and occurred following the transition to ICD-10 in the United States. Second, in order to evaluate differences between definitions among a subset of patients with higher acuity, we compared the definitions in a subset limited to admitted or transferred patients only. Analyzes were conducted using the survey package (version 4.0)^27^ in R, version 4.0.5 (R Foundation for Statistical Computing, Vienna, Austria, https://www.R-project.org/).

## Results

### Patient Inclusion

**Figure 1.**
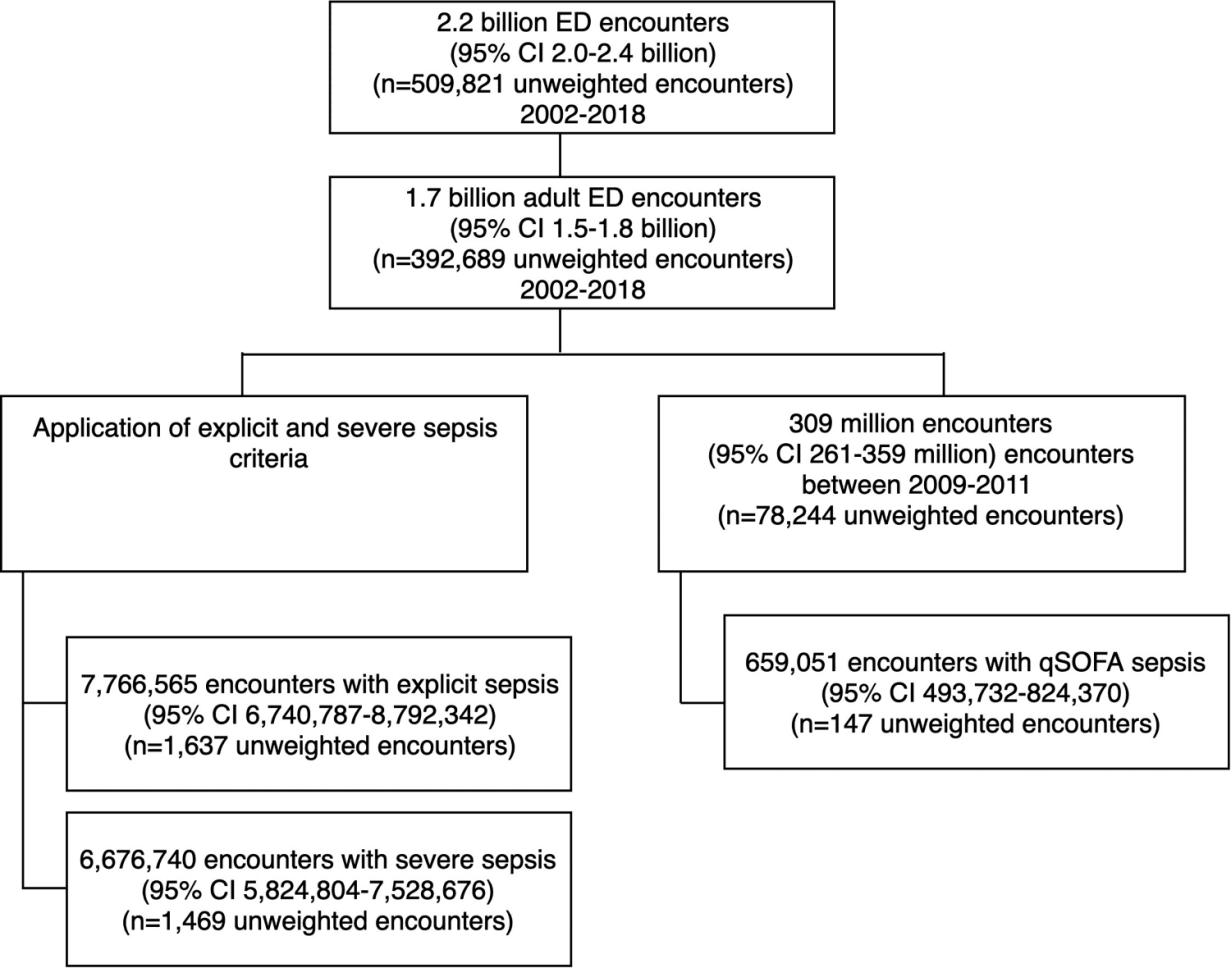
Encounter inclusion and identification of patient cohorts.

ED, emergency department; CI, confidence interval; SIRS, systemic inflammatory response syndrome; qSOFA, quick sequential organ failure assessment.

During the 17-year period, an estimated survey-weighted 2.2 billion encounters (95% CI 2.0-2.4 billion) occurred in US EDs. This figure was derived from 509 821 unweighted encounters within the NHAMCS dataset. Of these, 23.2% (95% CI 22.3%-24.0%) were classified as pediatric and excluded. Among all adult ED encounters, the mean age was 46.0 years (95% CI 45.7-46.3 years) and 43.0% (95% CI 42.7%-43.3%) were male. Inclusion criteria are detailed in Figure 1. Assessments were missing in the following proportions: heart rate in 6.4%, respiratory rate in 4.7% (for years 2007 onwards), temperature in 6.3%, blood pressure in 4.0%, and GCS in 67.9% (for the years 2009-2011).

### Sepsis Estimates Using Each Criterion

Sepsis estimates for each of the three applied criteria are provided in Table 1. The highest estimate was obtained using the explicit sepsis criteria, which averaged 0.45 million encounters per year. The lowest early estimate for sepsis was obtained using the qSOFA criteria, which averaged 0.22 million encounters per year. When evaluating as population-adjusted rates, there was overlap in the 95% CI among all three criteria, with the highest point estimate identified among encounters meeting explicit sepsis criteria, occurring in an age-adjusted 18.6 per 10 000 adults per year.

**Table 1.** Estimates for Sepsis Using Each Criteria.

Variable	Explicit sepsis	Severe sepsis, Wang/Angus criteria	qSOFA score ≥2, with infection
Number, millions (95% CI)	7.77 (6.74-8.79)	6.67 (5.82-7.52)	0.65 (0.49-0.82)
Percent of all encounters	0.47 (0.42-0.51)	0.40 (0.36-0.44)	0.21 (0.17-0.25)
Number of unweighted included encounters	1637	1469	147
Number of years	17	17	3
Yearly estimate, millions	0.45	0.39	0.22
Population adjusted yearly estimate, per 10 000 adults (95% CI); crude	19.5 (12.9-26.3)	16.8 (10.6-23.1)	9.3 (5.8-12.9)
Population adjusted yearly estimate, per 10 000 adults (95% CI); age adjusted^a^	18.6 (13.8-23.5)	16.1 (11.6-20.5)	8.9 (3.0-14.8)

Abbreviations: CI, confidence interval; SIRS, systemic inflammatory response syndrome; qSOFA, quick sequential organ failure assessment.

^a^ Adjusted for 2000 US Census data.

### Characteristics, Vital Signs, and Treatments of Sepsis Cohorts

Characteristics of the three groups identified using the different sepsis criteria are provided in Table 2. All groups had a slightly higher proportion of older adults (>65 years), the majority were of White race and non-Hispanic ethnicity, and were publicly insured. A slight majority in all three groups were brought to the hospital by EMS. Encounters meeting the severe sepsis criteria had a high proportion with hypotension (53.6%) relative to the other cohorts (Table 3). There was a high proportion with tachypnea (89.4%) and altered mental status (51.4%) in the group identified using qSOFA. Complete blood counts were obtained for the majority of patients using all criteria. Blood lactate was unavailable for the qSOFA cohort and was obtained in a slightly higher proportion of encounters with explicit sepsis compared to severe sepsis. The highest rate of admission/transfer was identified among encounters with explicit sepsis, though confidence intervals overlapped with encounters identified using qSOFA.

**Table 2.** Demographics of Sepsis Encounters Using Each Criteria. Numbers Within the Table Represent Survey-Weighted Percents among Encounters Meeting Each Listed Criteria.

Variable	Explicit sepsis	Severe sepsis, Wang/Angus criteria	qSOFA score ≥2, with infection
	Survey weighted percent (95% CI)	Survey weighted percent (95% CI)	Survey weighted percent (95% CI)
Age			
Adult (18-6 years)	40.0 (36.8-43.3)	44.4 (40.6-48.2)	37.4 (26.4-48.4)
Older adult (>65 years)	60.0 (56.7-63.2)	55.6 (51.8-59.4)	62.6 (51.6-73.6)
Male sex	51.6 (48-55.2)	51.0 (47.5-54.4)	42.5 (33.2-51.8)
Race			
White	76.2 (73.1-79.4)	80.0 (76.8-83.2)	81.9 (72.9-90.9)
Black	19.0 (16.2-21.9)	15.5 (12.6-18.4)	^b^
Other	4.7 (3.1-6.3)	4.5 (3.0-6.0)	^b^
Non-Hispanic ethnicity^a^	90.5 (88.0-92.9)	91.8 (89.3-94.4)	93.2 (89.0-97.5)
Insurance			
Private	14.8 (12.7-16.8)	17.3 (14.5-20.1)	^b^
Public	74.8 (71.8-77.8)	70.3 (66.6-74)	73.6 (63.9-83.3)
Other/not specified	10.4 (8.1-12.8)	12.4 (9.4-15.3)	^b^
Metropolitan status area	85.5 (79.4-91.6)	84.4 (78.5-90.4)	79.4 (67.1-91.6)
Geographic region			
Northeast	20.0 (15.3-24.6)	20.2 (14.5-25.9)	^b^
Midwest	21.8 (15.6-28.1)	24.2 (18.7-29.8)	27.6 (16.1-39.1)
South	35.0 (28.4-41.6)	34.7 (28.7-40.7)	38.3 (26.2-50.5)
West	23.2 (18.6-27.8)	20.8 (16.3-25.4)	^b^
Arrival by EMS	54.7 (50.9-58.5)	53.4 (48.3-58.5)	58.6 (47.5-69.7)

Abbreviations: qSOFA, quick sequential organ failure assessment; EMS, emergency medical services; CI, confidence interval.

^a^ Available for year 2007 onwards.

^b^ Unable to derive estimates due to cell size restrictions in NHAMCS.

**Table 3.** Clinical Characteristics and Treatments Provided for Each Studied Sepsis Definition. Numbers Within the Table Represent Survey-Weighted Percents among Encounters Meeting Each Listed Criteria.

Variable	Explicit sepsis	Severe sepsis, Wang/Angus criteria	qSOFA score ≥2, with infection
	Survey weighted percent (95% CI)	Survey weighted percent (95% CI)	Survey weighted percent (95% CI)
Fever or hypothermia	33.2 (29.9-36.5)	46.7 (42.5-50.8)	24.6 (16.6-32.6)
Tachycardia	50.3 (47.5-53.1)	36.5 (32.9-40.1)	50.7 (40.4-61.0)
Hypotension	13.1 (10.7-15.4)	53.6 (48.4-58.8)	27.8 (18.0-37.5)
Tachypnea^a^	55.5 (51.3-59.7)	48.0 (43.2-52.7)	89.4 (83-95.9)
Hypoxemia	6.4 (4.9-7.9)	7.4 (5.1-9.6)	^b^
GCS <15^a^	^b^	^b^	51.5 (41.6-61.3)
Testing			
Blood culture	53.0 (48.9-57.2)	32.7 (29.1-36.3)	45.1 (36.0-54.2)
Complete blood count	85.9 (82.8-89.0)	82.8 (79.6-86.1)	87.2 (80.0-94.4)
Lactate^a^	32.3 (26.4-38.2)	20.5 (14.8-26.1)	^b^
Urinalysis	63.1 (59.9-66.4)	52.6 (48.4-56.8)	62.8 (52.2-73.3)
Any radiography^a^	82.5 (79.2-85.8)	78.7 (74.9-82.5)	75.6 (67.5-83.8)
Treatment factors			
Given antibiotics	72.1 (68.2-76)	50.9 (46.8-55)	78.3 (70.4-86.3)
Given pressors	7.5 (5.8-9.2)	10.0 (8.0-11.9)	^b^
Given intravenous fluids	78.2 (74.8-81.6)	75.0 (72.0-78.1)	83.6 (75.7-91.6)
Endotracheal intubation	4.0 (2.7-5.3)	17.6 (15.2-20)	^b^
Disposition			
Admit/Transferred	91.8 (89.6-94)	75.2 (71.6-78.8)	81.7 (73.4-90.1)
Discharged	5.0 (3.4-6.6)	20.9 (17.6-24.1)	^b^
Other	3.2 (1.7-4.8)	3.9 (2.3-5.5)	^b^
Outcome			
In-hospital mortality	10.2 (7.9-12.5)	9 (6.9-11.1)	^b^

Abbreviations: qSOFA, quick sequential organ failure assessment; EMS, emergency medical services; CI, confidence interval.

^a^ GCS only available for years 2009-2011, imaging for year 2005 onwards, respiratory rate for years 2007 onwards, and lactate from 2012 onwards.

^b^ Unable to derive estimates due to cell size restrictions in NHAMCS.

### Longitudinal Trends in Sepsis Cohorts

**Figure 2.**
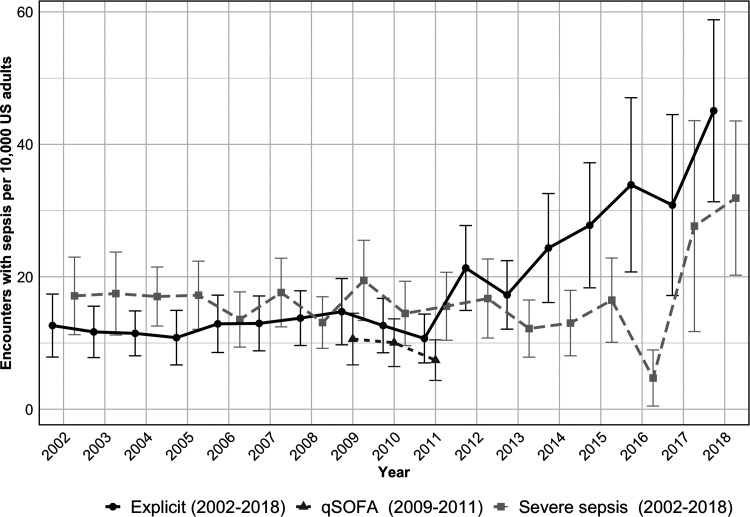
Population-adjusted trends of sepsis criteria. qSOFA, quick Sepsis Related Organ Failure Assessment.

When adjusted for the US adult population, the explicit sepsis criteria demonstrated a rise in encounters starting in 2011 (Figure 2). Estimates for explicit sepsis increased from 7.9 to 31.3 per 10 000 population between 2002 to 2018. Encounters for severe sepsis demonstrated a similar rise in encounters though starting at a later time point (2016). Severe sepsis was identified in 17.1 encounters per 10 000 adults in 2002 and in 31.9 encounters per 10 000 in 2018. Yearly encounters of qSOFA sepsis, available for only three years, overlapped with explicit and severe sepsis estimates during the available time frame.

### Cohort Overlap

**Figure 3.**
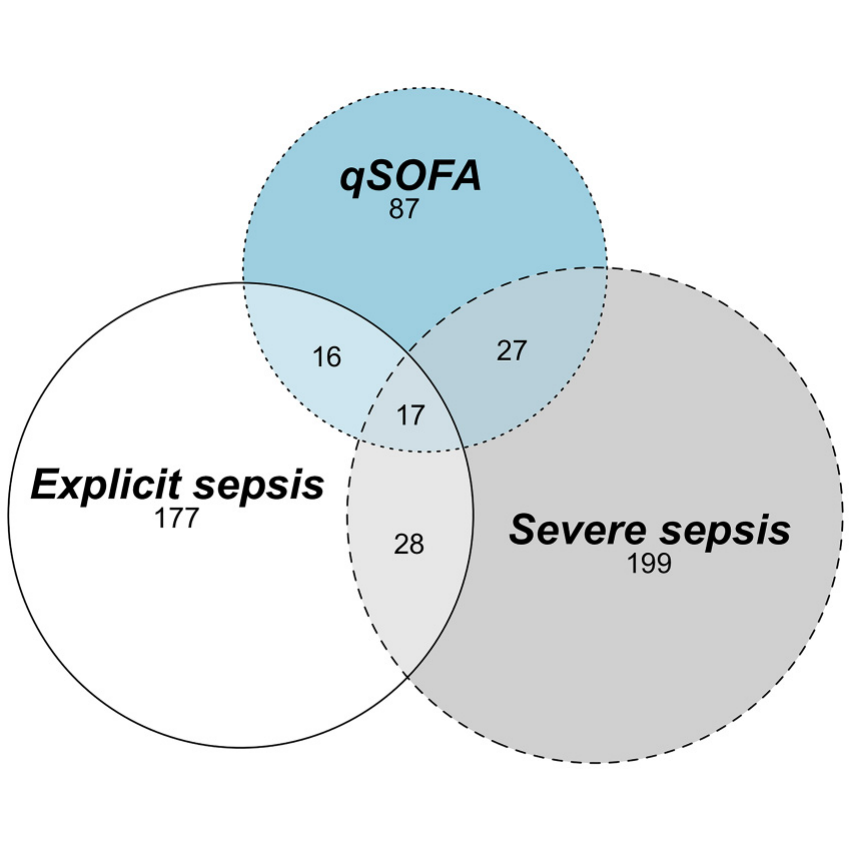
Overlap of unweighted encounters meeting varying sepsis criteria. qSOFA, quick Sepsis Related Organ Failure Assessment.

Of 551 unweighted encounters meeting at least one set of sepsis criteria, overlap was identified in 88 (16%). Seventeen encounters (3%) met all three criteria (Figure 3). Among the 17 encounters meeting all three criteria, six had pneumonia and four had urinary tract infections. Most (n = 12) patients who met all three criteria were greater than 65 years of age.

### Post-hoc Analyzes

When comparing sepsis definitions from the years 2016 to 2018, higher rates of both explicit sepsis and severe sepsis were identified. The age-adjusted rate of explicit sepsis was 33.1 cases per 10 000 and 19.3 per 10 000 for severe sepsis (Supplementary Table 3). The explicit sepsis cohort demonstrated a trend toward high acuity disease, as evidenced by rates of blood culture and lactate acquisition and antibiotic provision (Supplementary Table 4). 90.9% (95% CI 85.8%-96.0%) patients with explicit sepsis and 84.2% (76.2%-92.3%) patients with severe sepsis were admitted to the hospital. When inspecting raw counts, 378 had explicit sepsis, 184 had severe sepsis, and 55 met both sets of criteria (10.8% of patients meeting at least one criteria).

In a second *post hoc* analysis comparing only admitted patients, rates of sepsis were slightly lower for each definition compared to the primary analysis (Supplementary Table 5). The explicit sepsis cohort again demonstrated a higher rate of blood culture performance and antibiotic use compared to the other definitions (Supplementary Table 6). Overlap remained minimal in this subset, with 17 of 439 (4%) meeting all three sets of criteria.

## Discussion

### Summary of Key Findings

We conducted a repeated cross-sectional analysis of a multicenter national stratified survey dataset of ED encounters to compare estimates of different sets of sepsis criteria to assess their degree of overlap, and to evaluate their trends over time. Though yearly estimates were similar when evaluating the three studied sets of criteria (ranging from 220 000 to 450 000 per year), there was minimal overlap between the studied sepsis criteria. We identified variation in proxy markers for patient acuity among the three sets of criteria, including with respect to hospitalization and use of antibiotics. Estimates for explicit and severe sepsis demonstrated an increase when trended horizontally.

### Comparison to Recent Literature

The findings from this study fit into the context of several recent studies which have investigated the administrative coding of sepsis for research and epidemiological surveillance^4–6,11,15–19^ and expand upon the existing knowledge of the ED presentation sepsis in the United States. In particular, this study expands on prior work using the NHAMCS dataset investigating the ED management of sepsis^5,6,9^ by utilizing recent coding schema (ie, post-transition to ICD-10 codes), as well as facilitating a comparison of these criteria to each other by evaluating their overlap and by providing clinical data to demonstrate differences in acuity between the cohorts.

### Comparison of Present Findings to ED Sepsis Estimates in Adults

Previous efforts to utilize the NHAMCS dataset to evaluate sepsis have suggested varying estimates of sepsis. Wang et al*.* using the NHAMCS datasets for the years 2001 to 2004, identified 571 000 annual case visits with severe sepsis, which compares to our reported rate of 390 000 visits/year^5^ Our reported rate for explicit sepsis (450 000/year) is higher than that reported by with those of Filbin et al*.* who utilized a similar methodology for the years 1994 to 2009 and identified 260 000 US ED cases of sepsis per year; this corroborates with our evaluation of longitudinal trends which demonstrate an increase in the use of these diagnosis codes over time^6^ Notably, the use of explicit code-based approaches may have limited sensitivity. In one audit of patients with clinically documented severe sepsis, one-fourth of patients with disease did not have diagnosis codes for septicemia or bacteremia.^28^

Our results suggest that defining sepsis using qSOFA score generates estimates which are lower than those obtained using explicit and severe sepsis criteria. Importantly, qSOFA has demonstrated mixed performance in clinical practice. While one study of patients admitted to the intensive care unit suggested superior performance characteristics compared to SIRS characteristics,^21^ other studies have suggested its value may be more limited, particularly in the prehospital, ED, and non-intensive care inpatient settings.^29–31^

### Longitudinal Trends

We noted important longitudinal trends for the severe sepsis and explicit sepsis estimates. The severe sepsis cohort notably demonstrated a rise in encounters that coincided with the transition between ICD-9 to ICD-10 in the US, suggesting that these criteria may be increasingly unstable in the ICD-10 era. The use of explicit diagnosis codes, first published as ICD-9 codes in 2002,^32^ has been reported to have higher specificity compared to other strategies.^6,15,16^ A US based study which evaluated clinical (ie Sepsis-3) criteria, explicit criteria, and severe sepsis criteria among adults admitted for sepsis for the years 2009 to 2014 suggested that severe sepsis was identified more frequently compared to explicit sepsis,^33^ a finding compatible with the initial study period in the present work. A Spanish study investigated the use of explicit sepsis codes between 2006 to 2011 and reported a gradual increase in identified encounters over time, with a greater proportion of patients having organ dysfunction and in-hospital mortality.^16^ In contrast, our study suggests relatively stable use of explicit codes prior to 2011 in the US, with rates of use subsequently tripling in the ensuing seven years. This change occurred prior to the transition from ICD-9 to ICD-10 in the US, and prior to guidelines for sepsis published by the United States Centers for Medicare and Medicaid Services (CMS) in 2015,^34^ suggesting that the change was driven by some other cause.

### Overlap of Sepsis Definitions

We identified that overlap was minimal between the explicit, severe sepsis and qSOFA criteria in this study. This finding, which was also noted in *post-hoc* analyzes, demonstrates an inherent challenge in the accurate identification of sepsis from administrative datasets. The differences in identified cohorts using these three sets of criteria can be partially explained by the evolution of clinical sepsis definitions over the past two decades. Sepsis previously consisted of the presence of SIRS with evidence of infection, according to criteria developed by the American College of Chest Physicians and Society of Critical Care Medicine in 1992.^1^ Severe sepsis, intended to indicate organ dysfunction in the presence of infection, required lactatemia, oliguria, or changes to mental status.^34^ The use of diagnosis codes from administrative datasets may provide a limited means to reliably identify encounters with organ dysfunction, especially when limited to diagnosis codes placed in the ED.^5,6^ In general, we found that the severe sepsis criteria had evidence of lower acuity disease, including lower hospital admissions and lower use of antibiotics compared to patients identified using explicit criteria. Rates of endotracheal intubation and hypotension were high in this cohort as they were part of the criteria. The role of the qSOFA score and its overlap with other sepsis definitions warrants further investigation. For example, one prehospital study demonstrated a low sensitivity of prehospital assessment of qSOFA score for detecting sepsis/septic shock in the ED,^30^ while other studies have demonstrated that the qSOFA score demonstrates good performance for outcomes including admission and in-hospital mortality.^21–23^

### Comparison to Work Utilizing a Clinical Reference Standard

Less work has compared administrative definitions of sepsis (such as the explicit and severe sepsis definitions described in the present study) to clinical definitions of this condition. The definition of “severe sepsis” as described as organ dysfunction associated with organ dysfunction, hypoperfusion, or hypotension was outlined in the Sepsis 1 definitions in 1991.^1^ While organ dysfunction is a component of the Sepsis-2^35^ and Sepsis-3^3^ criteria, the term “severe sepsis” is not used in these definitions. In contrast, the administrative definition of explicit definition of sepsis is generally less specific and could be refer to patients meeting Sepsis-1, 2, or 3 clinical definitions of sepsis. A US study comparing explicit and implicit codes to Sepsis-3 criteria suggested that they had poor sensitivity to the clinical definition, with explicit codes having a sensitivity of 32% and a combination of implicit and explicit codes having a sensitivity of 66%.^33^ One study, performed among patients admitted to a German university hospital, compared overlap between sepsis codes and noted that the explicit sepsis definition had low sensitivity (25%) for any sepsis outcome (ie the sepsis-1 definitions of sepsis and severe sepsis) compared to a reference standard of manual chart review.^10^ Though the implicit definition of sepsis had a higher sensitivity for this outcome (59%), it was still low. Finally, a recent study that compared use of sepsis definitions in the ED to a reference standard noted that a higher proportion of patients with Sepsis-3 criteria met criteria for severe sepsis at the time of hospital discharge compared to explicit sepsis.^11^

### Limitations

Our findings are subject to limitations inherent to NHAMCS, including errors with documentation, abstraction, and coding.^36,37^ Our assessments for abnormalities in vital signs were limited only to triage vital signs only. Our findings were limited only to ED stays. For example, if antibiotics were initiated after hospitalization following a short ED stay, this would not be identified in the dataset. We were only able to evaluate the qSOFA score-based definition of sepsis for a limited time frame, creating challenges with respect to comparisons to the other two definitions. While NHAMCS has data on whether certain tests were performed (such as a complete blood count), data pertaining to test results (such as the white blood cell count) are not available in the dataset. As such, we could not compare differences in lab values between definitions. We used established definitions for the identification of sepsis from administrative datasets over a long period of time, during which clinical definitions of sepsis continued to evolve. Data on GCS were present in fewer than half of patients. It is unknown if missing data with respect to GCS are missing at random, though potentially the majority of encounters with missing GCS had a normal score. Nonetheless, these findings are indicative of the challenges inherent to the validation of any retrospective data source, where data with mental status may not be readily available. Finally, we were unable to compare our results to a reference standard, such as the Sepsis-3 criteria, given the lack of needed data within NHAMCS for this outcome.

## Conclusion

We performed a cross-sectional study to assess and compare rates of sepsis using multiple criteria. We identified substantial variability with respect to estimates of sepsis. Furthermore, while overlapping with respect to annual rates, different sets of criteria for sepsis demonstrated varying rates of clinical testing, antibiotic use, and disposition, with minimal overlap between unweighted encounters. These findings inform efforts aimed at developing approaches to identify reliable estimates of sepsis from US hospital settings.

## Supplemental Material

sj-docx-1-jic-10.1177_08850666221080060 - Supplemental material for Varying Estimates of Sepsis among Adults Presenting to US Emergency Departments: Estimates from a National Dataset from 2002-2018 Click here for additional data file. Supplemental material, sj-docx-1-jic-10.1177_08850666221080060 for Varying Estimates of Sepsis among Adults Presenting to US Emergency Departments: Estimates from a National Dataset from 2002-2018 by Sriram Ramgopal, Christopher M Horvat and Mark D Adler in Journal of Intensive Care Medicine

sj-docx-2-jic-10.1177_08850666221080060 - Supplemental material for Varying Estimates of Sepsis among Adults Presenting to US Emergency Departments: Estimates from a National Dataset from 2002-2018 Click here for additional data file. Supplemental material, sj-docx-2-jic-10.1177_08850666221080060 for Varying Estimates of Sepsis among Adults Presenting to US Emergency Departments: Estimates from a National Dataset from 2002-2018 by Sriram Ramgopal, Christopher M Horvat and Mark D Adler in Journal of Intensive Care Medicine

sj-docx-3-jic-10.1177_08850666221080060 - Supplemental material for Varying Estimates of Sepsis among Adults Presenting to US Emergency Departments: Estimates from a National Dataset from 2002-2018 Click here for additional data file. Supplemental material, sj-docx-3-jic-10.1177_08850666221080060 for Varying Estimates of Sepsis among Adults Presenting to US Emergency Departments: Estimates from a National Dataset from 2002-2018 by Sriram Ramgopal, Christopher M Horvat and Mark D Adler in Journal of Intensive Care Medicine

sj-docx-4-jic-10.1177_08850666221080060 - Supplemental material for Varying Estimates of Sepsis among Adults Presenting to US Emergency Departments: Estimates from a National Dataset from 2002-2018 Click here for additional data file. Supplemental material, sj-docx-4-jic-10.1177_08850666221080060 for Varying Estimates of Sepsis among Adults Presenting to US Emergency Departments: Estimates from a National Dataset from 2002-2018 by Sriram Ramgopal, Christopher M Horvat and Mark D Adler in Journal of Intensive Care Medicine

sj-docx-5-jic-10.1177_08850666221080060 - Supplemental material for Varying Estimates of Sepsis among Adults Presenting to US Emergency Departments: Estimates from a National Dataset from 2002-2018 Click here for additional data file. Supplemental material, sj-docx-5-jic-10.1177_08850666221080060 for Varying Estimates of Sepsis among Adults Presenting to US Emergency Departments: Estimates from a National Dataset from 2002-2018 by Sriram Ramgopal, Christopher M Horvat and Mark D Adler in Journal of Intensive Care Medicine

sj-docx-6-jic-10.1177_08850666221080060 - Supplemental material for Varying Estimates of Sepsis among Adults Presenting to US Emergency Departments: Estimates from a National Dataset from 2002-2018 Click here for additional data file. Supplemental material, sj-docx-6-jic-10.1177_08850666221080060 for Varying Estimates of Sepsis among Adults Presenting to US Emergency Departments: Estimates from a National Dataset from 2002-2018 by Sriram Ramgopal, Christopher M Horvat and Mark D Adler in Journal of Intensive Care Medicine
