# Supplementary material for: Varying Estimates of Sepsis among Adults Presenting to US Emergency Departments: Estimates from a National Dataset from 2002-2018
Source: J Intensive Care Med. 2022 Feb 28;37(11):1451–9. doi: 10.1177/08850666221080060 (PMC9548922; doi:10.1177/08850666221080060)
Supplement: sj-docx-1-jic-10.1177_08850666221080060 - Supplemental material for Varying Estimates of Sepsis among Adults Presenting to US Emergency Departments: Estimates from a National Dataset from 2002-2018 [file sj-docx-1-jic-10.1177_08850666221080060.docx]

**Supplementary Table 1.** Sepsis definitions applied

| **Criteria** | **Definition** | **Reference(s)** |
| --- | --- | --- |
| Explicit sepsis definition | Explicit documentation of sepsis as an ICD-9/ICD-10 diagnosis (Supplementary Table 2) | Filbin, 2014 [1] (applied to NHAMCS) |
| Severe sepsis, Wang, criteria | Evidence of infection based on ICD-9/ICD-10 coding (Supplementary Table 2) or the presence of fever (≥38.0 °C) or hypothermia (<36.0 °C)  AND  Evidence of organ dysfunction based on diagnosis coding (Supplementary Table 2), procedure of endotracheal intubation, or hypotension (systolic pressure ≤90 mmHg) | Angus, 2001 [2] (original criteria)  Wang, 2007 [3] (modification to NHAMCS) |
| qSOFA score ≥2, with infection | At least two of the following: a) GCS <15; b) Respiratory rate ≥22/minute; c) Systolic blood pressure ≤100 mm/Hg  AND  Evidence of infection based on ICD-9/ICD-10 coding (Supplementary Table 2) or the presence of fever (≥38.0 °C) or hypothermia (<36.0 °C) | *qSOFA definition:*  Seymour, 2016 [4]  *Codes for infection*  Angus, 2001 [2]  Wang, 2007 [3] |

ICD-9, International Classification of Disease, ninth revision; SIRS, systemic inflammatory response syndrome; qSOFA, quick Sepsis Related Organ Failure Assessment

**References**

[1] Filbin MR, Arias SA, Camargo CA, Barche A, Pallin DJ. Sepsis visits and antibiotic utilization in U.S. Emergency departments. Crit Care Med 2014;42:528–35. https://doi.org/10.1097/CCM.0000000000000037.

[2] Angus DC, Linde-Zwirble WT, Lidicker J, Clermont G, Carcillo J, Pinsky MR. Epidemiology of severe sepsis in the United States: Analysis of incidence, outcome, and associated costs of care. Crit Care Med 2001;29:1303–10. https://doi.org/10.1097/00003246-200107000-00002.

[3] Wang HE, Shapiro NI, Angus DC, Yealy DM. National estimates of severe sepsis in United States emergency departments. Crit Care Med 2007;35:1928–36. https://doi.org/10.1097/01.CCM.0000277043.85378.C1.

[4] Seymour CW, Liu VX, Iwashyna TJ, Brunkhorst FM, Rea TD, Scherag A, et al. Assessment of clinical criteria for sepsis for the third international consensus definitions for sepsis and septic shock (sepsis-3). JAMA - J Am Med Assoc 2016;315:762–74. https://doi.org/10.1001/jama.2016.0288.
