# Supplementary material for: Varying Estimates of Sepsis among Adults Presenting to US Emergency Departments: Estimates from a National Dataset from 2002-2018
Source: J Intensive Care Med. 2022 Feb 28;37(11):1451–9. doi: 10.1177/08850666221080060 (PMC9548922; doi:10.1177/08850666221080060)
Supplement: sj-docx-2-jic-10.1177_08850666221080060 - Supplemental material for Varying Estimates of Sepsis among Adults Presenting to US Emergency Departments: Estimates from a National Dataset from 2002-2018 [file sj-docx-2-jic-10.1177_08850666221080060.docx]

**Supplemental Table 2.** ICD codes for infection, organ dysfunction, and explicit sepsis.

| **Infection** |
| --- |
| **ICD-9:** 001, 002, 003, 004, 005, 008, 009, 010, 011, 012, 013, 014, 015, 016, 017, 018, 020, 021, 022, 023, 024, 025, 026, 027, 030, 031, 032, 033, 034, 035, 036, 037, 038, 039, 040, 041, 090, 091, 092, 093, 094, 095, 096, 097, 098, 100, 101, 102, 103, 104, 110, 111, 112, 114, 115, 116, 117 118, 320, 322, 324, 325, 420, 421, 451, 461, 462, 463, 464, 465, 481, 482, 485, 486, 491.21, 494, 510, 513, 540, 541, 542, 562.01, 562.03, 562.11, 562.13, 566, 567, 5695, 569.83, 572.0, 572.1, 575.0, 590, 597, 599.0, 601, 614, 615, 616, 681, 682, 683, 686, 711.0, 730, 790.7, 958.3, 996.6, 998.5, 999.3  **ICD-10:** A00.0, A00.1, A00.9, A01.00, A01.1, A01.2, A01.3, A01.4, A02.0, A02.1, A02.20, A02.21, A02.22, A02.23, A02.24, A02.29, A02.8, A02.9, A03.0, A03.1, A03.2, A03.3, A03.8, A03.9, A05.0, A05.1, A05.2, A05.3, A05.4, A05.5, A05.8, A05.9, A04.0, A04.1, A04.2, A04.3, A04.4, A04.5, A04.6, A04.71, A04.72, A04.8, A04.9, A08.0, A08.11, A08.19, A08.2, A08.31, A08.32, A08.39, A08.8, A09, A15.6, A15.7, A15.0, A15.5, A15.4, A15.8, A17.0, A17.1, A17.81, A17.82, A17.89, A17.9, A18.31, A18.32, A18.39, A18.01, A18.02, A18.03, A18.10, A18.11, A18.12, A18.13, A18.14, A18.15, A18.16, A18.17, A18.18, A18.2, A18.4, A18.50, A18.51, A18.52, A18.53, A18.54, A18.59, A18.6, A18.7, A18.81, A18.84, A18.85, A18.89, A19.2, A19.8, A19.9, A20.0, A20.1, A20.2, A20.7, A20.8, A20.9, A21.0, A21.1, A21.2, A21.3, A21.7, A21.8, A21.9, A22.0, A22.1, A22.2, A22.7, A22.8, A22.9, A23.0, A23.1, A23.2, A23.3, A23.8, A23.9, A24.0, A24.3, A24.9, A25.0, A25.1, A25.9, A26.7, A26.8, A26.9, A28.0, A28.8, A28.9, A32.11, A32.12, A32.7, A32.81, A32.89, A32.9, A30.0, A30.1, A30.3, A30.5, A30.8, A30.9, A31.0, A31.1, A31.2, A31.8, A31.9, A36.0, A36.1, A36.2, A36.3, A36.81, A36.82, A36.83, A36.84, A36.85, A36.86, A36.89, A36.9, A37.00, A37.10, A37.80, A37.90, A38.9, J02.0, J03.00, A46, A39.0, A39.1, A39.4, A39.50, A39.51, A39.52, A39.53, A39.81, A39.82, A39.83, A39.89, A39.9, A35, A40.3, A40.9, A41.01, A41.02, A41.1, A41.2, A41.3, A41.4, A41.50, A41.51, A41.52, A41.53, A41.59, A41.89, A41.9, A42.0, A42.1, A42.2, A42.81, A42.82, A42.89, A42.9, A43.8, A43.9, B47.1, B47.9, L08.1, A48.0, A48.3, A48.51, A48.52, A48.8, K90.81, M60.009, A49.3, B95.0, B95.1, B95.2, B95.3, B95.4, B95.5, B95.61, B95.62, B95.7, B95.8, B96.0, B96.1, B96.20, B96.21, B96.22, B96.23, B96.29, B96.3, B96.4, B96.5, B96.6, B96.7, B96.81, B96.89, A50.09, A50.1, A50.2, A50.31, A50.40, A50.41, A50.42, A50.45, A50.49, A50.52, A50.57, A50.59, A50.6, A50.7, A50.9, A51.0, A51.1, A51.2, A51.31, A51.32, A51.39, A51.41, A51.43, A51.45, A51.46, A51.49, A51.5, A52.00, A52.01, A52.02, A52.03, A52.06, A52.09, A52.11, A52.13, A52.14, A52.15, A52.17, A52.19, A52.2, A52.3, A52.71, A52.72, A52.73, A52.74, A52.75, A52.76, A52.77, A52.78, A52.79, A52.8, A52.9, A53.0, A53.9, A54.00, A54.01, A54.03, A54.21, A54.22, A54.23, A54.24, A54.29, A54.31, A54.32, A54.33, A54.39, A54.40, A54.41, A54.42, A54.49, A54.5, A54.6, A54.81, A54.83, A54.85, A54.86, A54.89, A27.0, A27.81, A27.89, A27.9, A69.0, A69.1, A66.0, A66.1, A66.2, A66.3, A66.4, A66.5, A66.6, A66.7, A66.8, A66.9, A67.0, A67.1, A67.2, A67.3, A67.9, A65, A69.8, A69.9, B35.0, B35.1, B35.2, B35.3, B35.4, B35.5, B35.6, B35.8, B35.9, B36.0, B36.1, B36.2, B36.3, B36.8, B36.9, B37.0, B37.1, B37.2, B37.3, B37.42, B37.49, B37.5, B37.6, B37.7, B37.81, B37.82, B37.83, B37.84, B37.89, B37.9, B38.0, B38.1, B38.2, B38.3, B38.4, B38.89, B38.9, B39.2, B39.3, B39.4, B39.5, B39.9, G02, H32, I32, I39, J17, B40.9, B41.0, B41.9, B48.0, B42.0, B42.1, B42.7, B42.9, B43.9, B44.9, B45.0, B45.7, B45.9, B46.9, B47.0, B48.1, B48.2, B48.8, B49, G00.0, G00.1, G00.2, G00.3, G00.8, G00.9, G01, G04.2, G03.0, G03.1, G03.8, G03.9, G06.0, G06.1, G06.2, G08, I30.0, I30.8, I30.9, I33.0, I33.9, I80.00, I80.10, I80.209, I80.219, I80.3, I80.8, I80.9, J01.00, J01.10, J01.20, J01.30, J01.40, J01.90, J02.9, J03.90, J04.0, J04.10, J04.11, J04.2, J04.30, J04.31, J05.0, J05.10, J05.11, J06.0, J06.9, J13, J18.1, A48.1, J14, J15.0, J15.1, J15.20, J15.211, J15.212, J15.29, J15.3, J15.4, J15.5, J15.6, J15.8, J15.9, J18.0, J18.9, J44.1, J47.1, J47.9, J86.0, J86.9, J85.0, J85.1, J85.2, J85.3, K35.2, K35.3, K35.80, K35.89, K37, K36, K57.12, K57.13, K57.32, K57.33, K61.0, K61.1, K61.3, K65.0, K65.1, K65.2, K65.3, K65.4, K65.8, K65.9, K67, K68.12, K68.19, K68.9, K63.0, K63.1, K75.0, K75.1, K81.0, N10, N11.0, N11.8, N12, N15.1, N15.9, N16, N28.84, N28.85, N28.86, N34.0, N34.1, N34.2, N34.3, N39.0, N41.0, N41.1, N41.2, N41.3, N41.4, N41.8, N41.9, N51, N70.01, N70.02, N70.03, N70.11, N70.12, N70.13, N70.91, N70.92, N70.93, N73.0, N73.1, N73.2, N73.3, N73.4, N73.6, N73.8, N73.9, N71.0, N71.1, N71.9, N72, N75.0, N75.1, N75.9, N76.0, N76.1, N76.2, N76.3, N76.4, N76.5, N76.6, N76.81, N76.89, N77.0, N77.1, L03.019, L03.029, L03.039, L03.049, K12.2, L03.119, L03.129, L03.211, L03.212, L03.213, L03.221, L03.222, L03.317, L03.319, L03.329, L03.811, L03.818, L03.891, L03.898, L03.90, L03.91, L04.9, E83.2, L08.0, L08.89, L08.9, L88, L98.0, M00.00, M00.019, M00.029, M00.039, nM0.0049, M00.059, M00.069, M00.079, M00.08, M00.09, M00.10, M00.119, M00.129, M00.139, M00.149, M00.159, M00.169, M00.179, M00.18, M00.19, M00.20, M00.219, M00.229, M00.239, M00.249, M00.259, M00.269, M00.279, M00.28, M00.29, M00.80, M00.819, M00.829, M00.839, M00.849, M00.859, M00.869, M00.879, M00.88, M00.89, M00.9, M46.20, M46.30, M86.10, M86.119, M86.129, M86.139, M86.149, M86.159, M86.169, M86.179, M86.18, M86.19, M86.20, M86.219, M86.229, M86.239, M86.249, M86.259, M86.269, M86.279, M86.28, M86.29, M86.60, M86.619, M86.629, M86.639, M86.642, M86.659, M86.669, M86.679, M86.68, M86.69, M86.9, M89.60, M89.619, M89.629, M89.639, M89.649, M89.659, M89.669, M89.679, M89.68, M89.69, M90.80, M90.819, M90.829, M90.839, M90.849, M90.859, M90.869, M90.879, M90.88, M90.89, R78.81, T79.8XXA, T82.6XXA, T82.7XXA, T83.510A, T83.511A, T83.512A, T83.518A, T83.590A, T83.591A, T83.592A, T83.593A, T83.598A, T83.61XA, T83.62XA, T83.69XA, T84.50XA, T84.60XA, T84.7XXA, T85.71XA, T85.730A, T85.731A, T85.732A, T85.733A, T85.734A, T85.735A, T85.738A, T85.79XA, K68.11, T81.4XXA, T80.211A, T80.212A, T80.219A, T80.22XA, T80.29XA, T88.0XXA |
| **Organ dysfunction** |
| **ICD-9:** 785.5, 458, 458.8, 458.9, 286.6, 286.9, 287.4, 287.5, 570, 573.4, 293, 348.3, 584, 518.8, 786.03, 799.1  **ICD-10:** D65, D68.8, D68.9, D69.51, D69.59, D69.6, F05, F06.0, F06.1, F06.2, F063.0, F06.4, F06.8, F53, G93.40, G93.41, G93.49, I67.83, I95.1, I95.2, I95.3, I95.81, I9589, I95.89, I95.9, I95.89, I95.9, J80, J96.00, J96.10, J96.20, J96.90, J98.4, R06.03, K72.00, K76.2, K76.3, N17.0, N17.1, N17.2, N17.8, N17.9, R57.0, R57.1, R57.8, R579, R65.21, R06.81, R09.2 |
| **Explicit sepsis** |
| **ICD-9:** 038, 785.52, 995.91, 995.92  **ICD-10**: A40.3, A40.9, A410.1, A410.2, A41.1, A41.2, A41.3, A41.4, A41.50, A41.51, A41.52, A41.53, A41.59, A4189, R65.21, A41.9, R65.20 |
