# Supplementary material for: Varying Estimates of Sepsis among Adults Presenting to US Emergency Departments: Estimates from a National Dataset from 2002-2018
Source: J Intensive Care Med. 2022 Feb 28;37(11):1451–9. doi: 10.1177/08850666221080060 (PMC9548922; doi:10.1177/08850666221080060)
Supplement: sj-docx-3-jic-10.1177_08850666221080060 - Supplemental material for Varying Estimates of Sepsis among Adults Presenting to US Emergency Departments: Estimates from a National Dataset from 2002-2018 [file sj-docx-3-jic-10.1177_08850666221080060.docx]

**Supplementary Table 3.** Estimate for sepsis using each criteria as a post-hoc analysis limited to the years 2016-2018 only.

| **Variable** | **Explicit sepsis** | **Severe sepsis, Wang/Angus criteria** |
| --- | --- | --- |
| Number, millions (95% CI) | 2.8 (2.0-2.4) | 1.6 (1.0-2.2) |
| Percent of all encounters | 0.87 (0.70-1.04) | 0.51 (0.37-0.65) |
| Number of unweighted included encounters |  |  |
| Number of years | 3 | 3 |
| Yearly estimate, millions | 0.92 | 0.54 |
| Population adjusted yearly estimate, per 10,000 adults (95% CI); crude | 36.6 (23.1-50.2) | 21.5 (10.9-32.1) |
| Population adjusted yearly estimate, per 10,000 adults (95% CI); age adjusted* | 33.1 (15.9-50.3) | 19.3 (5.7-32.9) |

CI, confidence interval; SIRS, systemic inflammatory response syndrome; qSOFA, quick sequential organ failure assessment

*Adjusted for 2000 US Census data
