# Supplementary material for: Varying Estimates of Sepsis among Adults Presenting to US Emergency Departments: Estimates from a National Dataset from 2002-2018
Source: J Intensive Care Med. 2022 Feb 28;37(11):1451–9. doi: 10.1177/08850666221080060 (PMC9548922; doi:10.1177/08850666221080060)
Supplement: sj-docx-4-jic-10.1177_08850666221080060 - Supplemental material for Varying Estimates of Sepsis among Adults Presenting to US Emergency Departments: Estimates from a National Dataset from 2002-2018 [file sj-docx-4-jic-10.1177_08850666221080060.docx]

**Supplementary Table 4.** Demographics and treatment characteristics for each sepsis criteria as a post-hoc analysis limited to the years 2016-2018 only. Numbers within the table represent survey-weighted percents among encounters meeting each listed criteria.

| **Variable** | **Explicit sepsis** | **Severe sepsis, Wang/Angus criteria** |
| --- | --- | --- |
|  | **Survey weighted percent (95% CI)** | **Survey weighted percent (95% CI)** |
| **Demographics** | | |
| Age |  |  |
| Adult (18-6 years) | 41.9 (36.1-47.7) | 37.1 (27.6-46.6) |
| Older adult (>65 years) | 58.1 (52.3-63.9) | 62.9 (53.4-72.4) |
| Male sex | 49.0 (42.8-55.2) | 53.2 (43.7-62.6) |
| Race |  |  |
| White | 76.1 (69.6-82.6) | 79.5 (70.4-88.6) |
| Black | 18.6 (13.0-24.3) | 16.7 (9.0-24.5) |
| Other | 5.3 (1.9-8.7) | ** |
| Non-Hispanic ethnicity* | 90.4 (85.7-95.1) | 89.5 (83.2-95.9) |
| Insurance |  |  |
| Private | 13.1 (9.4-16.8) | 12.4 (6.5-18.3) |
| Public | 75.5 (69.6-81.4) | 76.2 (65.9-86.6) |
| Other/not specified | 11.4 (6.5-16.2) | ** |
| Metropolitan status area | 83.0 (17.3-94.6) | 90.4 (81.8-99.0) |
| Geographic region |  |  |
| Northeast | 14.2 (6.4-22.0) | 24.9 (6.2-43.7) |
| Midwest | 23.7 (9.3-38.1) | 20.0 (5.6-34.4) |
| South | 36.1 (23.1-49.1) | 36.9 (20.6-53.1) |
| West | 25.9 (16.4-35.4) | 182. (6.8-29.7) |
| Arrival by EMS | 51.1 (45.2-57.1) | 51.1 (39.5-58.8) |
| **Clinical characteristics** | | |
| Fever or hypothermia | 25.5 (19.3-31.8) | 31.3 (22.0-40.6) |
| Tachycardia | 52.9 (47.8-57.9) | 38.3 (29.0-47.7) |
| Hypotension | 10.5 (6.5-14.4) | 27.7 (17.4-38.1) |
| Tachypnea* | 49.7 (42.5-56.8) | 49.7 (39.7-58.7) |
| Hypoxemia | ** | ** |
| **Testing** | | |
| Blood culture | 53.8 (46.5-61.2) | 34.9 (27.0-42.7) |
| Complete blood count | 83.9 (77.5-90.2) | 89.0 (82.2-95.8) |
| Lactate* | 41.2 (33.4-49.0) | 24.5 (15.3-33.6) |
| Urinalysis | 59.8 (52.9-66.8) | 53.3 (41.0-65.9) |
| Any radiography* | 85.1 (79.6-90.6) | 84.1 (77.7-90.4) |
| **Treatment factors** | | |
| Given antibiotics | 78.0 (70.9-85.2) | 52.7 (40.5-64.9) |
| Given pressors | ** | ** |
| Given intravenous fluids | 77.0 (70.1-84.0) | 75.2 (68.0-82.4) |
| Endotracheal intubation | ** | ** |
| **Disposition** | | |
| Admit/Transferred | 90.9 (85.8-96.0) | 84.2 (76.2-92.3) |
| Discharged | ** | ** |
| Other | ** | ** |
| **Outcome** | | |
| In-hospital mortality | ** | ** |

*Ethnicity for 2007 onwards, imaging for year 2005 onwards, respiratory rate for years 2007 onwards

**Unable to derive estimates due to cell size restrictions in NHAMCS.
