# Supplementary material for: Varying Estimates of Sepsis among Adults Presenting to US Emergency Departments: Estimates from a National Dataset from 2002-2018
Source: J Intensive Care Med. 2022 Feb 28;37(11):1451–9. doi: 10.1177/08850666221080060 (PMC9548922; doi:10.1177/08850666221080060)
Supplement: sj-docx-5-jic-10.1177_08850666221080060 - Supplemental material for Varying Estimates of Sepsis among Adults Presenting to US Emergency Departments: Estimates from a National Dataset from 2002-2018 [file sj-docx-5-jic-10.1177_08850666221080060.docx]

**Supplementary Table 5.** Estimate for sepsis using each criteria as a post-hoc analysis limited to admitted/transferred patients only.

| **Variable** | **Explicit sepsis** | **Severe sepsis, Wang/Angus criteria** | **qSOFA score ≥2, with infection** |
| --- | --- | --- | --- |
| Number, millions (95% CI) | 7.13 (6.14-8.11) | 5.02 (4.29-5.74) | 0.53 (0.39-0.69) |
| Percent of all encounters | 0.47 (0.42-0.51) | 0.40 (0.36-0.44) | 0.21 (0.17-0.25) |
| Number of unweighted included encounters | 1513 | 1059 | 119 |
| Number of years | 17 | 17 | 3 |
| Yearly estimate, millions | 042 | 0.29 | 0.18 |
| Population adjusted yearly estimate, per 10,000 adults (95% CI); crude | 18.0 (11.6-24.3) | 12.7 (7.4-17.9) | 7.6 (4.5-10.8) |
| Population adjusted yearly estimate, per 10,000 adults (95% CI); age adjusted* | 17.1 (12.5-21.7) | 11.9 (8.2-15.7) | 7.3 (2.2-12.4) |

CI, confidence interval; SIRS, systemic inflammatory response syndrome; qSOFA, quick sequential organ failure assessment

*Adjusted for 2000 US Census data
