# Supplementary material for: Varying Estimates of Sepsis among Adults Presenting to US Emergency Departments: Estimates from a National Dataset from 2002-2018
Source: J Intensive Care Med. 2022 Feb 28;37(11):1451–9. doi: 10.1177/08850666221080060 (PMC9548922; doi:10.1177/08850666221080060)
Supplement: sj-docx-6-jic-10.1177_08850666221080060 - Supplemental material for Varying Estimates of Sepsis among Adults Presenting to US Emergency Departments: Estimates from a National Dataset from 2002-2018 [file sj-docx-6-jic-10.1177_08850666221080060.docx]

**Supplementary Table 6.** Demographics and treatment characteristics for each sepsis criteria as a post-hoc analysis limited to admitted/transferred patients only. Numbers within the table represent survey-weighted percents among encounters meeting each listed criteria.

| **Variable** | **Explicit sepsis** | **Severe sepsis, Wang/Angus criteria** | **qSOFA score ≥2, with infection** |
| --- | --- | --- | --- |
|  | **Survey weighted percent (95% CI)** | **Survey weighted percent (95% CI)** | **Survey weighted percent (95% CI)** |
| **Demographics** | | | |
| Age |  |  |  |
| Adult (18-6 years) | 39.1 (35.8-42.3) | 39.2 (35.0-43.3) | 28.8 (18.0-39.5) |
| Older adult (>65 years) | 60.9 (57.7-64.2) | 60.8 (56.7-65) | 71.2 (60.5-82) |
| Male sex | 52.1 (48.4-55.9) | 45.3 (41.3-49.3) | 49.6 (39.1-60.1) |
| Race |  |  |  |
| White | 75.7 (72.2-79.1) | 80.4 (76.6-84.3) | 79.7 (69.4-90.1) |
| Black | 19.4 (16.3-22.5) | 14.7 (11.3-18) | ** |
| Other | 5.0 (3.3-6.7) | 4.9 (3.1-6.8) | ** |
| Non-Hispanic ethnicity | 90.8 (88.2-93.3) | 93.1 (90.6-95.7) |  |
| Insurance |  |  |  |
| Private | 14.7 (12.5-16.9) | 15 .0(11.8-18.2) | ** |
| Public | 75.7 (72.7-78.7) | 74.4 (70-78.7) | 80.2 (71.9-88.5) |
| Other/not specified | 9.6 (7.5-11.8) | 10.7 (7-14.3) | ** |
| Metropolitan status area | 85.9 (79.9-92.0) | 87.3 (82.3-92.3) | 80.1 (67.3-92.9) |
| Geographic region |  |  |  |
| Northeast | 19.7 (14.9-24.4) | 21.3 (14.1-28.5) | ** |
| Midwest | 21.3 (14.8-27.7) | 24.9 (18.9-30.9) | 29.1 (16.5-41.7) |
| South | 36.0 (29.0-43.0) | 33.8 (27.1-40.4) | 36.7 (23.6-49.8) |
| West | 23.0 (18.3-27.8) | 20.1 (15.3-24.8) | ** |
| Arrival by EMS | 56.1 (52.3-59.9) | 58.5 (52.9-64.1) | 67.7 (57-78.5) |
| **Clinical characteristics** | | | |
| Fever or hypothermia | 34 (30.4-37.6) | 45.1 (40.1-50.1) | 25.1 (16.7-33.6) |
| Tachycardia | 49.8 (46.9-52.6) | 38.6 (34.3-42.8) | 49.1 (38.6-59.6) |
| Hypotension | 12.5 (10.3-14.8) | 47.8 (41.8-53.7) | 30.4 (19.3-41.4) |
| Tachypnea* | 55.1 (51-59.3) | 49.4 (44.2-54.7) | 88.6 (80.9-96.4) |
| Hypoxemia | 6.6 (4.9-8.2) | 8.7 (6.2-11.2) | ** |
| **Testing** | | |  |
| Blood culture | 54.9 (50.7-59.1) | 39.5 (35.4-43.6) | 52.2 (41.4-62.9) |
| Complete blood count | 88.1 (85.3-90.9) | 90.2 (87.7-92.8) | 93.8 (89.5-98.0) |
| Lactate* | 34.1 (28.0-40.2) | 23.1 (16.3-29.9) | ** |
| Urinalysis | 63.9 (60.7-67.1) | 56.3 (51.4-61.2) | 62.7 (51.3-74.1) |
| Any radiography* | 84.5 (81.3-87.6) | 86.1 (82.5-89.6) | 83 (75.4-90.5) |
| **Treatment factors** | | |  |
| Given antibiotics | 73.8 (69.9-77.7) | 54.2 (49.2-59.1) | 81.4 (73.2-89.5) |
| Given pressors | 8.0 (6.1-9.8) | 11.8 (9.4-14.2) | ** |
| Given intravenous fluids | 80.6 (77.2-83.9) | 80.9 (77.6-84.1) | 87.0 (80.3-93.6) |
| Endotracheal intubation | 3.9 (2.5-5.3) | 20.9 (17.9-23.9) | ** |
| **Disposition** | | | |
| In-hospital mortality | 10.2 (8.1-12.3) | 9.7 (7.4-12) | ** |

*Ethnicity for 2007 onwards, imaging for year 2005 onwards, respiratory rate for years 2007 onwards

**Unable to derive estimates due to cell size restrictions in NHAMCS
